# Effect of Cytomegalovirus Co-Infection on Normalization of Selected T-Cell Subsets in Children with Perinatally Acquired HIV Infection Treated with Combination Antiretroviral Therapy

**DOI:** 10.1371/journal.pone.0120474

**Published:** 2015-03-20

**Authors:** Suad Kapetanovic, Lisa Aaron, Grace Montepiedra, Patricia Anthony, Kasalyn Thuvamontolrat, Savita Pahwa, Sandra Burchett, Adriana Weinberg, Andrea Kovacs, for the Pediatric AIDS Clinical Trials Group Protocol 366

**Affiliations:** 1 National Institutes of Health, National Institute of Mental Health, Bethesda MD, United States of America; 2 Center for Biostatistics in AIDS Research, Harvard School of Public Health, Boston MA, United States of America; 3 Maternal, Child and Adolescent Center for Infectious Diseases and Virology, University of Southern California Keck School of Medicine, Los Angeles CA, United States of America; 4 University of Miami Miller School of Medicine, Miami FL, United States of America; 5 Children’s Hospital Boston and Harvard Medical School, Boston MA, United States of America; 6 University of Colorado Denver, Denver CO, United States of America; University of Cape Town, SOUTH AFRICA

## Abstract

**Background:**

We examined the effect of cytomegalovirus (CMV) co-infection and viremia on reconstitution of selected CD4+ and CD8+ T-cell subsets in perinatally HIV-infected (PHIV+) children ≥ 1-year old who participated in a partially randomized, open-label, 96-week combination antiretroviral therapy (cART)-algorithm study.

**Methods:**

Participants were categorized as CMV-naïve, CMV-positive (CMV+) viremic, and CMV+ aviremic, based on blood, urine, or throat culture, CMV IgG and DNA polymerase chain reaction measured at baseline. At weeks 0, 12, 20 and 40, T-cell subsets including naïve (CD62L+CD45RA+; CD95-CD28+), activated (CD38+HLA-DR+) and terminally differentiated (CD62L-CD45RA+; CD95+CD28-) CD4+ and CD8+ T-cells were measured by flow cytometry.

**Results:**

Of the 107 participants included in the analysis, 14% were CMV+ viremic; 49% CMV+ aviremic; 37% CMV-naïve. In longitudinal adjusted models, compared with CMV+ status, baseline CMV-naïve status was significantly associated with faster recovery of CD8+CD62L+CD45RA+% and CD8+CD95-CD28+% and faster decrease of CD8+CD95+CD28-%, independent of HIV VL response to treatment, cART regimen and baseline CD4%. Surprisingly, CMV status did not have a significant impact on longitudinal trends in CD8+CD38+HLA-DR+%. CMV status did not have a significant impact on any CD4+ T-cell subsets.

**Conclusions:**

In this cohort of PHIV+ children, the normalization of naïve and terminally differentiated CD8+ T-cell subsets in response to cART was detrimentally affected by the presence of CMV co-infection. These findings may have implications for adjunctive treatment strategies targeting CMV co-infection in PHIV+ children, especially those that are now adults or reaching young adulthood and may have accelerated immunologic aging, increased opportunistic infections and aging diseases of the immune system.

## Introduction

At least 3.3 million infants and children are living with human immunodeficiency virus (HIV) infection worldwide and 260,000 new perinatal HIV infections occur each year [1]. With increased access to combination antiretroviral therapy (cART), prolonged survival and increasingly chronic nature of their illness, it is essential to better understand the various mechanisms that affect the course and management of perinatally acquired HIV infection. Following initiation of cART in perinatally HIV-infected (PHIV+) children, there is a recovery of immune function, including significant increase of CD4+ T-cell count and percentage, decrease in CD8+ T-cell percentage, decrease in count and percentage of activated CD8+CD38+HLA-DR+ T-cells and increase in percentage of naïve CD4+ and CD8+CD62L+CD45RA+ T-cells [2]. Yet, immune activation levels remain elevated and many important immunologic parameters do not return to normal levels, prompting questions about residual mechanisms affecting normalization of T-cell compartments even in the circumstance of successful suppression of HIV replication [2].

Cytomegalovirus (CMV) is a potent activator of CD8+ T cells in the general population, including HIV-co-infected individuals [3]. PHIV+ infants co-infected with CMV have higher CD8+ and lower CD4+ T-cell counts than HIV mono-infected children [4]. In a small cohort of HIV+ adults, valganciclovir was efficacious in suppressing CMV DNA to undetectable levels and reducing CD8+CD38+HLA-DR+% [5], a sensitive marker of T-cell activation [2, 6], suggesting not only that CMV replication contributes to immune activation in co-infected individuals, but also that pharmacologic suppression of CMV may help reverse this process [5]. Yet, these data may not be generalizable to PHIV+ children, whose patterns of immune reconstitution differ from those of adults. Thus, it is critically important to evaluate the impact of CMV co-infection on the recovery of a normal T-cell distribution of PHIV+ children in response to cART. Furthermore, it has been suggested that CMV may accelerate immunologic aging leading to early onset of diseases related to the aging process [7].

We evaluated longitudinal trends in percentages of selected T-cell phenotypes in a cohort of PHIV+ children with severe disease following a switch in ART regimen. We focused on activated (CD38+HLA-DR+); naïve (CD62L+CD45RA+; CD95-CD28+); and terminally differentiated (CD62L-CD45RA+; CD95+CD28-) [8–10] compartments of both CD4+ and CD8+ T-cells. We hypothesized that CMV co-infection and active CMV viremia would impact T-cell activation and percentages of naïve and memory T cell populations. Additional CD4+ and CD8+ T-cell phenotypes were included for explorative purposes, and a post-hoc sub-analysis was conducted to explore the potential effect of CMV-specific cell-mediated immunity (CMI) on T-cell reconstitution.

## Materials and Methods

### Study Design

This research was approved by the University of Southern California Health Sciences Institutional Review Board. This was a nested retrospective study within the Pediatric AIDS Clinical Trials Group (PACTG) Protocol 366 (ACTG 366). ACTG 366 enrollment occurred between May 1998 and January 2000 at 50 participating sites in the US and Puerto Rico. Written informed consents from the next of kin, caretakers, or guardians were obtained on behalf of the minors and children enrolled in the study, including written assent according to local institutional review board guidelines. Patient records and information were anonymized and de-identified prior to analysis. ACTG 366 is registered with ClinicalTrials.gov under the following registration number: NCT00000902.

ACTG 366 enrolled PHIV+ ART-experienced participants 6 months to 21 years old with severe disease defined as having HIV-1 plasma viral load (VL) >50,000 copies/mL; CD4+ count <200 cells/mm3, CD4% <15, or a 50% reduction in CD4% within 24 weeks of the start of the current ART regimen; growth failure; or CNS disease. Participants started a new cART regimen according to a pre-defined algorithm as previously described [11]. HIV VL and CD4/CD8 cell counts were measured at baseline, monthly for the first six months and bimonthly thereafter. Advanced T-cell phenotyping and CMV cell-mediated immunity (CMI) assays were performed at baseline and weeks 12, 20 and 40. All assays were performed at PACTG-certified laboratories.

Our analysis included participants older than one year; who had baseline plasma samples available for CMV testing; and had baseline data available for at least one of the T-cell phenotypes of interest. Virology and immunology testing was performed in National Institutes of Health, Division of AIDS (DAIDS) approved laboratories that participated in viral and immunology quality assurance programs.

### Assessment of Viral Load and Viral Load Response

HIV-1 plasma VL was measured using ACD-treated blood and Roche Diagnostics Amplicor 1.0. Viral response was defined using operational definitions from the original study [11]. Specifically, the participants were considered VL responders (VLRs) if their VLs decreased to <400 copies/mL from baseline to the average of weeks 12 and 16; partial-VLRs if they had VL ≥400 copies/mL, but had a reduction in VL of at least 0.75 log_10_ copies/mL; the remaining participants were considered non-VLRs and were taken off study within 8 weeks unless they were considered by study chairs to show clinical benefits.

### Assessment of T-Cell Phenotypes

Whole blood (1.5mL) was collected in tubes with EDTA as the anticoagulant. Samples were shipped at room temperature by priority overnight express mail to designated special immunology laboratories, and processed for immunophenotyping using a standardized protocol that was quality controlled across all laboratories as per NIAID/DAIDS Flow Cytometry Guidelines for 3 color flow cytometry for instrument calibration, isotype controls, instrument compensation and gating strategy [12]. The monoclonal antibody (mAb) panel included 3 reagent combinations of CD3/CD4/CD45, CD3/CD8/CD45, and CD3/CD19/CD45 for identification of CD4 and CD8 T cells. Extended phenotyping was performed with the mAb combinations CD45RA/CD62L/CD4; CD45RA/CD62L/CD8; HLA-DR/CD38/CD4; HLA/DR/CD38/CD8; CD28/CD95/CD4; CD28/CD95/CD8 using green (FITC), orange(PE) and red (ECD or perCP or PE-Cy5) fluorochromes depending on instrument configuration. This panel was designed to identify the following CD4 and CD8 T cell subsets: Naïve (CD45RA+CD62L+) or (CD28+CD95-); effector (CD45RA+CD62L-) or (CD28-CD95+); and activated (HLADR+CD38+). For gating, we ensured 98% to 99% of all events in the Isotype control cursor settings were in the lower left quadrant of the flow plot. Cursor settings were left untouched or adjusted in accordance with the guidelines for different mAb combinations, as shown in the Sample Analysis appendix of the IMPAACT/PACTG Laboratory Manual [12].

The current analysis focuses on the following subsets of markers: CD4+CD38+HLA-DR+% and CD8+CD38+HLA-DR+% (activated); CD4+CD62L+CD45RA+%, CD8+CD62L+CD45RA+%, CD4+CD95-CD28+% and CD8+CD95-CD28+% (naïve); and CD4+CD62L-CD45RA+%, CD8+CD62L-CD45RA+%, CD4+CD95+CD28-% and CD8+CD95+CD28-% (terminally differentiated). The remaining permutations of expressed CD38/HLA-DR, CD62L/CD45RA and CD95/CD28 on CD4+ and CD8+ T-cells were also recorded and explored.

### Assessment of CMV infection status

CMV status was assessed at study pre-entry using blood, urine, or throat culture, serology, PCR or antigen testing, and results recorded in the ACTG 366 database. Additionally, CMV IgG (GenWay Biotech Inc., San Diego, CA Cat.#: 40-052-115031) and DNA quantitation by PCR (Abbott, Des Plaines, IL) were measured using plasma stored at baseline. Participants with detectable CMV DNA (≥12 copies/mL) were considered CMV-positive (CMV+) viremic. Participants who were CMV IgG positive (≥ 1.2 IU/mL) or had any positive result recorded at pre-entry but did not have detectable CMV DNA at baseline were considered CMV+ aviremic. All children who were CMV IgG sero-negative and had negative results on DNA PCR and all other clinical diagnostic tests were considered CMV uninfected or CMV-naïve.

### CMV-Specific Cell-Mediated Immunity

Lymphocyte proliferation assays (LPA) were performed at the designated PACTG core immunology laboratories using overnight shipped blood as previously described [13]. The laboratories used the same lot of CMV cell lysate and mock-infected control antigens prepared in Dr. Weinberg’s laboratory as previously described [14] and a consensus inter-AIDS networks LPA protocol [15]. Stimulation indices ≥3 defined response.

### Statistical Methods

The primary predictor of interest was CMV positivity and viremia status at ACTG 366 study entry. The outcomes of interest were the percentages of T-cell phenotypes described above. Participant characteristics were compared by CMV status using Kruskal-Wallis tests, Mantel Haenszel Chi-Square tests, and Chi-Square tests as appropriate.

Associations between baseline CMV status and T-cell subset percentages at weeks 0, 12, 20, and 40 were assessed with linear mixed models adjusting for gender, age group (1–5 years, 6–12 years, >12 years), CD4% at entry, baseline ART regimen (cART with nelfinavir, cART without nelfinavir, not on cART), baseline HIV-1 RNA (log_10_ copies/ml), race/ethnicity (Hispanic, Black, White/other), VL response at week 16 (responder, partial-responder, non-responder, missing) and treatment arm (main effect and interaction with week). The models were adjusted for nelfinavir-containing regimens because of known *in vitro* evidence of nelfinavir-associated inhibition of CMV replication in a human endothelial cell line [16]. Interaction terms between CMV status and week were added to the models to assess if the pattern of change in immune markers differed by CMV status.

Additionally, a post-hoc explorative sub-analysis was conducted to determine if the presence of CMV-specific CMI may contribute to protection against the CMV-driven T-cell differentiation. This sub-analysis focused only on percentages of T-cell subsets that had been found to be associated with baseline CMV status in the multivariate models. Positive CMI response was defined as a SI greater than or equal to 3 at week 0, 12, 20 or 40.

Two-sided p-values less than 0.05 were considered statistically significant. To retain maximum sensitivity for identifying potential effects of CMV co-infection, there were no adjustments made for multiple testing. All analyses were done using SAS Version 9.2.

## Results

### Participant Characteristics

Of the 200 children enrolled in ACTG 366, 107 met the inclusion criteria outlined in the methods. Their median age was 7 years (interquartile range 4–10 years); 62 (58%) were black non-Hispanic; and 59 (55%) were males (Table 1). At entry (prior to study treatment), 98% of participants were on some type of ART, and forty (37%) participants were in CDC disease category C [17]. Forty-one percent were VLRs at week 16, 19% were partial VLRs, and 26% were non-VLRs.

**Table 1:** Characteristics by CMV Status among ACTG 366 Participants with Immune Activation Data.

Characteristic		CMV+ viremic (N = 15)	CMV+ aviremic (N = 52)	CMV-naïve (N = 40)	Total (N = 107)	P-value
Age (yrs), median (Q1, Q3)		4 (3, 9)	8 (5, 10)	6 (3, 9)	7 (4, 10)	0.026 (a)
Age (yrs), N (%)	1–5 years	11 (73%)	18 (35%)	19 (48%)	48 (45%)	0.431 (b)
	6–12 years	3 (20%)	28 (54%)	17 (43%)	48 (45%)	
	>12 years	1 (7%)	6 (12%)	4 (10%)	11 (10%)	
Gender, N (%)	Male	4 (27%)	36 (69%)	19 (48%)	59 (55%)	0.007 (c)
Race/Ethnicity, N (%)	Black Non-Hispanic	10 (67%)	26 (50%)	26 (65%)	62 (58%)	0.270 (c)
	Hispanic (regardless of race)	5 (33%)	18 (35%)	8 (20%)	31 (29%)	
	White Non-Hispanic/Other	0 (0%)	8 (15%)	6 (15%)	14 (13%)	
CDC Category “C” at entry, N (%)		6 (40%)	19 (37%)	15 (38%)	40 (37%)	0.970 (c)
Baseline HIV-1 RNA level (copies/ml), median (Q1,Q3)		79,725 (44,203, 157,089)	60,670.0 (27,030.5, 121,584.5)	57,598.0 (22,426.0, 152,533.5)	59,818 (24,807, 129,510)	0.524 (a)
Baseline HIV-1 RNA level (copies/ml), N (%)	<50,000	5 (33%)	21 (40%)	18 (45%)	44 (41%)	0.699 (b)
	>50,000–100,000	4 (27%)	15 (29%)	7 (18%)	26 (24%)	
	>100,000	6 (40%)	16 (31%)	15 (38%)	37 (35%)	
CDC immunologic category	No suppression	4 (27%)	14 (27%)	14 (35%)	32 (30%)	0.957 (b)
	Moderate suppression	5 (33%)	26 (50%)	9 (23%)	40 (37%)	
	Severe suppression	6 (40%)	12 (23%)	17 (43%)	35 (33%)	
CD4 percent at entry, median(Q1,Q3)		22 (12, 24)	17 (11, 24)	21.5 (9.0, 28.5)	19 (11, 27)	0.638 (a)
CD4 percent at entry, N (%)	0–<15	5 (36%)	22 (43%)	14 (35%)	41 (39%)	0.213 (b)
	15–25	6 (43%)	19 (37%)	10 (25%)	35 (33%)	
	> 25	3 (21%)	10 (20%)	16 (40%)	29 (28%)	
Antiretroviral regimen at entry, N (%)	cART with NFV	3 (20%)	16 (31%)	10 (25%)	29 (27%)	0.887 (c)
	cART without NFV	3 (20%)	12 (23%)	9 (23%)	24 (22%)	
	Not on cART	9 (60%)	24 (46%)	21 (53%)	54 (50%)	
On cART at entry, N (%)		6 (40%)	28 (54%)	19 (48%)	53 (50%)	0.607 (c)
On PIs at entry, N (%)		6 (40%)	24 (46%)	14 (35%)	44 (41%)	0.557 (c)
On NNRTIs at entry, N (%)		3 (20%)	8 (15%)	10 (25%)	21 (20%)	0.515 (c)
ACTG 366 treatment arm*	1A	3 (20%)	10 (19%)	9 (23%)	22 (21%)	0.641 (c)
	1B	5 (33%)	9 (17%)	7 (18%)	21 (20%)	
	2	3 (20%)	17 (33%)	8 (20%)	28 (26%)	
	3 & 4	4 (27%)	16 (31%)	16 (40%)	36 (34%)	
Viral load response at week 16	Response	4 (27%)	20 (38%)	20 (50%)	44 (41%)	0.368 (c)
	Partial response	2 (13%)	12 (23%)	6 (15%)	20 (19%)	
	Non-response	6 (40%)	15 (29%)	7 (18%)	28 (26%)	
	Missing	3 (20%)	5 (10%)	7 (18%)	15 (14%)	

(a) Kruskal-Wallis Test

(b) Mantel Haenszel Chi-Square Test

(c) Chi-Square Test

cART = combination antiretroviral therapy; NFV = nelfinavir; NNRTI = non-nucleoside reverse transcriptase inhibitor; PI = protease inhibitor

*The participants in the protocol ACTG 366 were cross-classified according to NNRTI and PI exposure histories into four groups: 1 = NNRTI-PI-; 2 = NNRTI-PI+; 3 = NNRTI+PI-; and 4 = NNRTI+PI+. Group 1 was subdivided into Group 1A and 1B, where participants were randomized to be switched to either 2 NNRTIs different from current therapy + nevirapine/nelfinavir combination or 2 new NNRTIs + nevirapine/ritonavir combination. Group 2 was switched to 1 new NNRTI + nevirapine + nelfinavir + ritonavir. Groups 3 and 4 were switched to 2 new NNRTIs + nelfinavir + ritonavir. Enrollment occurred between May 1998 and January 2000 at 50 US sites. The original publication provides more detail on treatment algorithm and participant treatment histories [11].

The adjusted relationships of baseline CD4% counts and VL treatment responses with key T-cell subsets of interest are shown in Table 2 (for CD4+ subsets) and Table 3 (for CD8+ subsets). Higher CD4% at entry was significantly associated with higher CD4+CD62L+CD45RA+% and CD8+CD62L+CD45RA+% (naïve); higher CD4+CD95-CD28+% and CD8+CD95-CD28+% (naïve) and lower CD4+CD38+HLA-DR+% and CD8+CD38+HLA-DR+% (activated); lower CD4+CD95+CD28-% and CD8+CD95+CD28-% (terminally differentiated); and lower CD4+CD62L+CD45RA+% and CD8+CD62L+CD45RA+% (naïve) (p≤0.011). HIV-1 VL response at week 16 was not associated with changes of the T-cell subsets over time.

**Table 2:** Multivariate model estimates^1^ for selected CD4+ T-cell subtypes vs. selected key covariates.

		CD4+ subset									
		CD38+HLA-DR+		CD62L+CD45RA+		CD95-CD28+		CD95+CD28-		CD62L-CD45RA+	
Covariate		Est.	p	Est.	p	Est.	p	Est.	p	Est.	p
**Week**		−0.18	0.019	0.30	<0.001	0.10	0.26	−0.03	0.68	−0.02	0.48
**CMV status**			0.58		0.86		0.67		0.70		0.08
	**CMV+ viremic vs. naïve**	−4.30	0.32	2.76	0.60	−3.06	0.55	4.16	0.40	−1.30	0.27
	**CMV+ aviremic vs. naïve**	−0.16	0.96	0.95	0.79	1.60	0.65	0.58	0.86	−1.75	0.029
Week *CMV status			0.08		0.49		0.42		0.54		0.67
	Week *CMV+ viremic	0.26	0.041	−0.15	0.36	−0.19	0.19	0.14	0.28	0.04	0.38
	Week *CMV+ aviremic	−0.01	0.92	−0.12	0.28	−0.04	0.63	0.02	0.85	0.02	0.60
**CD4% at entry**		−0.78	<0.001	1.16	<0.001	1.28	<0.001	−0.81	<0.001	0.00	0.96

“Est.” = Estimate; “p” = p-value; “Week” = treatment week of the ACTG 366 clinical trial

^1^Estimates are adjusted for gender, age group (1–5yrs, 6–12 yrs, >12 yrs), baseline ARV regimen (cART with NFV, cART without NFV, no cART), HIV-1 RNA (log10cp/ml), race/ethnicity (Hispanic, Black, White/other), viral load response at week 16 (responder, partial-responder, non-responder, missing), treatment arm (1a, 1b, 2, ¾) main effect and interaction with week, in addition to covariates listed in table.

**Table 3:** Multivariate model estimates^1^ for selected CD8+ T-cell subtypes vs. selected key covariates.

		CD8+ subset									
		CD38+HLA-DR+		CD62L+CD45RA+		CD95-CD28+		CD95+CD28-		CD62L-CD45RA+	
Covariate		Est.	p	Est.	p	Est.	p	Est.	p	Est.	p
**Week**		−0.19	0.08	0.30	<0.001	0.20	0.011	−0.20	0.05	−0.05	0.45
**CMV status**			0.46		0.029		0.33		0.038		0.049
	**CMV+ viremic vs. naïve**	4.32	0.44	−8.79	0.06	−4.89	0.24	12.38	0.016	5.51	0.17
	**CMV+ aviremic vs. naïve**	4.40	0.25	−7.59	0.018	−3.47	0.22	5.49	0.11	6.71	0.018
Week *CMV status			0.41		0.025		0.004		0.003		0.38
	Week *CMV+ viremic	0.22	0.21	−0.23	0.10	−0.32	0.013	0.43	0.010	0.16	0.17
	Week *CMV+ aviremic	0.10	0.37	−0.24	0.008	−0.25	0.002	0.33	0.002	0.02	0.75
**CD4% at entry**		−0.63	<0.001	0.81	<0.001	0.20	0.011	−0.69	<0.001	−0.26	0.05

“Est.” = Estimate; “p” = p-value; “Week” = treatment week of the ACTG 366 clinical trial.

### Participant CMV Status at Study Baseline

Among the 107 participants, 15 (14%) were CMV+ viremic; 52 (49%) were CMV+ aviremic; and 40 (37%) were CMV-naïve. CMV+ aviremic participants were older (median age = 8 years) than CMV-naïve (6 years) and CMV+ viremic (4 years) participants (p = 0.026). CMV+ aviremic participants were more likely to be male (69%) than CMV-naïve (48%) and CMV+ viremic participants (27%) (p = 0.007). Other demographic and clinical characteristics were similarly distributed across the three CMV categories. (Table 1) In addition, there were no significant differences in demographic and HIV disease characteristics between all CMV+ and naïve participants. Among CMV+ viremic patients, the median CMV viral load was 38 cp/mL (range = 12–76,241).

### Relationship between CMV status and Percentages of T-Cell Phenotypes

**Fig 1:**
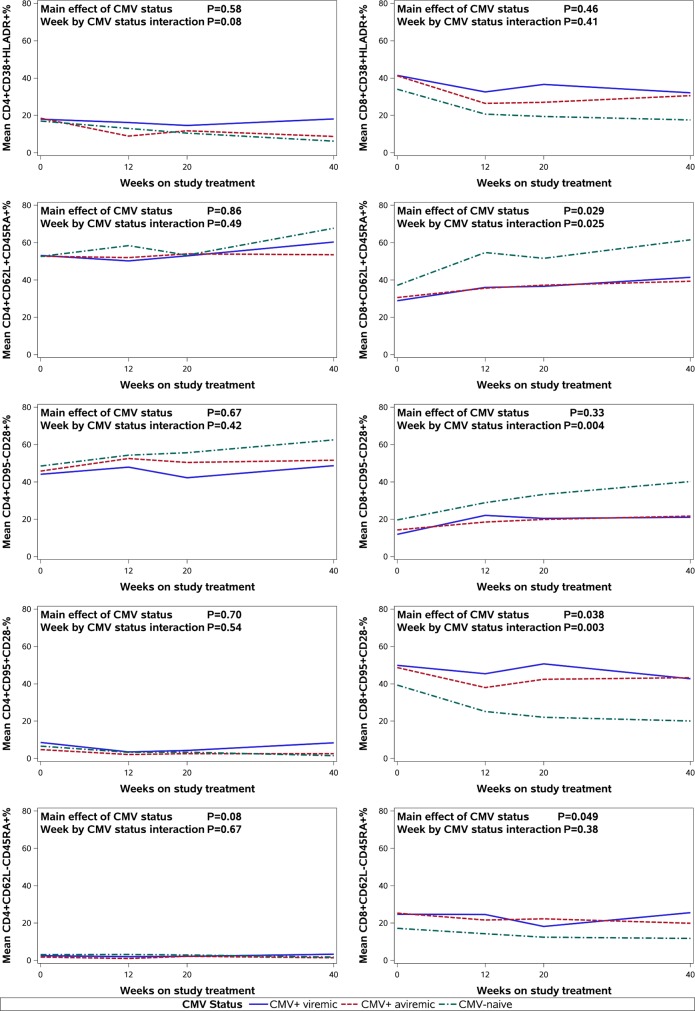
Mean T-cell Phenotype Percentages by CMV Status and Week. This figure shows trends in reconstitution of selected CD4+ and CD8+ T-cell phenotypes in response to cART by cytomegalovirus (CMV) co-infection status and study week in the ACTG 366. *Left column (top to bottom)*: The rate of change in the percentage of activated CD4+ T-cells (i.e., CD4+CD38+HLA-DR+%) was marginally significant by the CMV status. Neither naïve (i.e., CD4+CD62L+CD45RA+% and CD4+CD95-CD28+%) or terminally differentiated effector CD4+ T-cells (i.e., CD4+CD95+CD28- and CD4+CD62L-CD45RA+) were significantly affected by CMV co-infection or viremia status. *Right column (top to bottom)*: The rate of change in the percentage of activated CD8+ T-cells (i.e., CD8+CD38+HLA-DR+%) was not significantly affected by the CMV status. The increase in both CD8+ naïve subsets (i.e., CD8+CD62L+CD45RA+% and CD8+CD95-CD28+%) in response to cART was significantly slower in CMV+ viremic and aviremic groups compared to CMV-naïve group. The decrease in one of the terminally differentiated effector subsets of CD8+ T-cells (i.e., CD8+CD95+CD28-%) in response to cART was significantly slower in CMV+ viremic and aviremic groups compared to CMV-naïve group. The rate of change in the percentage of the other terminally differentiated effector CD8+ subset (i.e., CD8+CD62L-CD45RA+) in response to cART was not significantly affected by CMV co-infection or viremia status.

The kinetics of T-cell subsets of interest are depicted in the Fig. 1 by CMV status and week. (See S1 Table for a detailed summary.) Tables 2 and 3 show the longitudinal model estimates for the kinetics of the CD4+ (Table 2) and CD8+ (Table 3) T-cell subsets of interest versus selected key covariates—CMV status, CMV status by week interaction, and baseline CD4%. Models were also adjusted for age, gender, race/ethnicity, baseline HIV-1 RNA, treatment arm, treatment arm by week interaction, ART regimen, and HIV-1 VL response at study week 16.

### Activated CD4+ and CD8+ T-cells (CD38+HLA-DR+)

Although observed mean CD4+CD38+HLA-DR+% was similar in the 3 CMV categories at baseline, it was relatively higher in the CMV+ viremic group compared to the other 2 groups at weeks 12, 20, and 40 (Fig. 1; S1 Table). The multivariate model (Table 2) indicated that the rate of change in CD4+CD38+HLA-DR+% (activated) was marginally significant by CMV status (p = 0.08 for CMV status by week interaction). This observation was explained by the pairwise comparison, which showed significant differences between the CMV+ viremic group, who did not have a decrease in CD4+CD38+HLA-DR+% (activated) over time, and the CMV-naïve group who did show a decline in CD4 activation (p = 0.04; Table 2).

Observed mean CD8+CD38+HLA-DR+% (activated) was highest in the CMV+ viremic group and lowest in the CMV-naïve group at all time-points (Fig. 1; S1 Table), but these differences were not significant in the multivariate model (Table 3).

### Naïve CD4+ and CD8+ T-cells (CD62L+CD45RA+ and CD95-CD28+)

Mean CD4+CD62L+CD45RA+% (naïve) was similar in all three CMV categories (53%) at baseline (Fig. 1; S1 Table) and did not vary significantly by CMV status at baseline or over time in the multivariate model (Table 2).

At baseline, mean CD8+CD62L+CD45RA+% (naïve) was < 31% in the two CMV+ groups and 37% in CMV-naïve participants (p = 0.11; Fig. 1; S1 Table). In the multivariate model (Table 3), baseline CD8+CD62L+CD45RA+% (naïve) was significantly lower in CMV+ aviremic compared with CMV-naïve (p = 0.018) and marginally lower in the CMV+ viremic participants compared with CMV-naïve (p = 0.06). CD8+CD62L+CD45RA+ (naïve) percentages increased over time in all three groups and were persistently higher in the CMV-naïve compared with CMV+ groups (Fig. 1; S1 Table). The increase of CD8+CD62L+CD45RA+% (naïve) over time was significantly lower for CMV+ aviremic compared to CMV-naïve participants (p = 0.008) and marginally significantly lower for CMV+ viremic participants (p = 0.10; Table 3).

Mean CD4+CD95-CD28+% (naïve) was highest in the CMV-naïve and lowest in the CMV+ viremic group at all time-points (Fig. 1; S1 Table), but there were no significant differences by CMV status at baseline or over time in the multivariate model (Table 2).

Mean CD8+CD95-CD28+% (naïve) was highest in the CMV-naïve group at baseline and all time-points. The baseline differences in CD8+CD95-CD28+% (naïve) did not vary significantly by CMV status, but their increase over time was significantly lower in CMV+ viremic and aviremic compared to CMV-naïve participants (p = 0.004 for overall CMV status interaction effect, p = 0.013 for CMV+ viremic, p = 0.002 for CMV+ aviremic; Table 3).

### Terminally Differentiated Effector CD4+ and CD8+ T-cells (CD95+CD28- and CD62L-CD45RA+)

CMV+ viremic participants had the highest mean CD4+CD95+CD28-% (terminally differentiated) at all time-points (Fig. 1; S1 Table), but there were no significant differences in CD4+CD95+CD28-% (terminally differentiated) by CMV status in the multivariate model (Table 2).

In contrast at baseline, CMV+ viremic and CMV+ aviremic patients had similar percentages of CD8+CD95+CD28- (terminally differentiated) T cells (50% and 49% respectively) which were significantly higher than for the CMV-naïve participants (39%; p = 0.038). (Table 3; Fig. 1; S1 Table) Similarly, CMV had a significant effect on the CD8+CD95+CD28-% (terminally differentiated) following treatment (p = 0.016; Table 3), with significantly higher levels noted in both CMV+ viremic and aviremic compared to the CMV-naïve participants at weeks 12, 20 and 40. (Fig. 1; S1 Table) Furthermore, the decrease of CD8+CD95+CD28-% (terminally differentiated) over time was lower for CMV+ compared to CMV-naïve participants (p≤0.003). (Table 3)

CD4+CD62L-CD45RA+% (terminally differentiated) did not differ significantly between the three groups at any time-points (Fig. 1; S1 Table). In the multivariate model, baseline CD4+CD62L-CD45RA+% (terminally differentiated) was marginally significant by CMV status (p = 0.08 for overall main effect of CMV status) with the CMV+ aviremic group having lower CD4+CD62L-CD45RA+% (terminally differentiated) than the CMV-naïve group (p = 0.03; Table 2).

CMV+ participants had higher mean CD8+CD62L-CD45RA+% (terminally differentiated) at all four time-points than the CMV-naïve group (Fig. 1; S1 Table). The CMV+ aviremic group had higher mean CD8+CD62L-CD45RA+% (terminally differentiated) than the CMV-naïve group (p = 0.049 for overall main effect of CMV status, p = 0.018 for CMV+ aviremic; Table 3). However, the rate of change was not significantly different between the three groups (CMV status interaction effect overall p = 0.38; Table 3).

### The Relationship between CMV CMI and T-Cell Subset Changes in Response to cART

**Fig 2:**
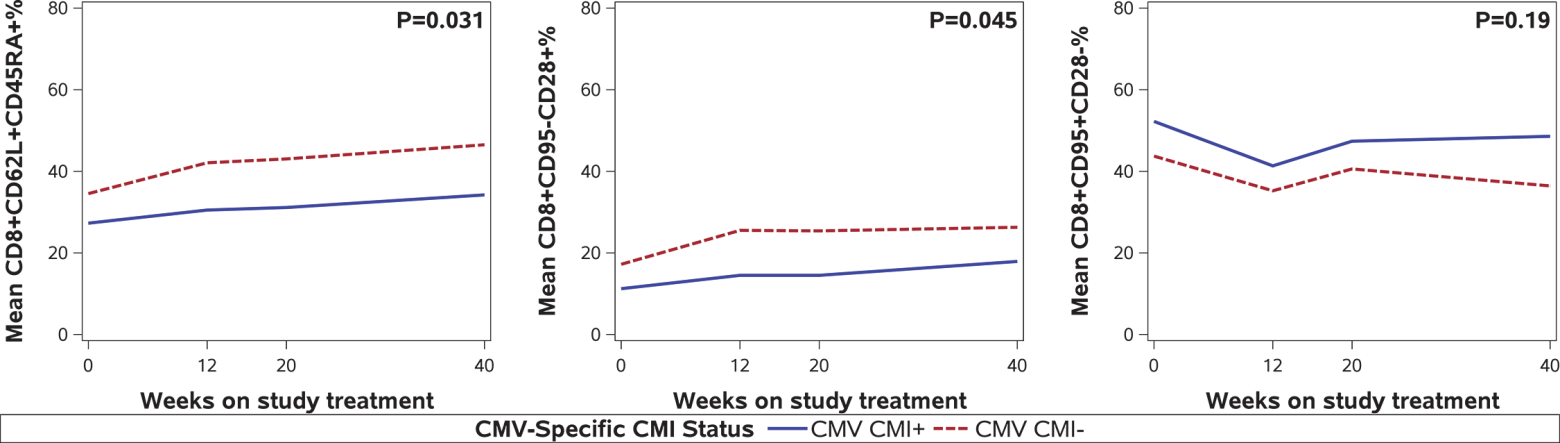
Effect of CMV-specific cell-mediated immunity (CMI) on normalization of selected T-cell subsets in CMV+ participants. This figure shows the relationship between CMV-specific cell-mediated immunity (CMI) and changes over time in three selected CD8+ T-cell subsets in response to cART among 103 eligible participants with available CMV CMI results. In linear mixed effects models, CMV CMI+ status was significantly associated with lower mean CD8+CD62L+CD45RA+% (naïve) (p = 0.03) and CD8+CD95-CD28+% (naïve) (p = 0.045), but not with CD8+CD95+CD28-% (terminally differentiated) (p = 0.19).

To determine if the presence of CMV-specific CMI may contribute to protection against the CMV-driven T-cell differentiation, we investigated the association of CMV CMI+ with the kinetics of differentiated and naïve T cells of CMV+ subjects. Of the 107 participants included in the analysis, 103 had CMV CMI data at one or more time points, and 63 of them were CMV+ (50 aviremic and 13 viremic). Of the 63 CMV+ participants, 33 (52%) had positive CMV CMI at one or more visits (CMV CMI+), and 30 (48%) had persistently negative CMV CMI (CMV CMI-). CMV+ aviremic participants were more likely to be CMI+ than CMV+ viremic patients (60% vs. 23%; p = 0.03). In linear mixed effects models, CMV CMI+ status was significantly associated with lower mean CD8+CD62L+CD45RA+% (naïve) (p = 0.03) and CD8+CD95-CD28+% (naïve) (p = 0.045), but not with CD8+CD95+CD28-% (terminally differentiated) (p = 0.19). (Fig. 2) The effect of CMV viremia status on this association could not be evaluated due to small number of CMV+ viremic participants with detectable CMV CMI.

## Discussion

We examined the effect of CMV co-infection and presence of CMV viremia on longitudinal reconstitution of CD4+ and CD8+ T-cell subsets in a cohort of ART-exposed PHIV+ children with severe disease who initiated a more aggressive cART regimen. The general effect of CMV co-infection status was most notable in the CD8+ population of T-cells with little impact on the CD4+ T-cell populations. CMV co-infection was significantly associated with lower percentages of naïve CD8+CD62L+CD45RA+ and CD8+CD95-CD28+ T-cells and higher percentage of terminally differentiated CD8+CD95+CD28- T-cells both at baseline and in their longitudinal trends. These associations were independent from the HIV VL response at week 16 and remained significant after adjusting for other important covariates, including treatment arm, baseline VL and baseline CD4% suggesting an independent role of CMV co-infection on the normalization of CD8+ T-cell population in PHIV+ children. Additionally, these data may help explain the noted increase in the development of opportunistic infections previously reported in children co-infected with CMV and HIV [4].

In contrast to studies in adults, our study did not find a significant impact of CMV status on activated CD8+ T-cells, as baseline CMV status was not associated with longitudinal trends in CD8+CD38+HLA-DR+%. This may be because PHIV+ infants and children acquire primary CMV infection while they have an immature immune system that is already suppressed by HIV and thus not allowing for the normal immune response of T-cell activation and other immunologic responses necessary to combat this primary infection whereas HIV infected adults in general are CMV-infected prior to their HIV infection. Alternatively, as implied by previous studies [18–20] CD38+HLA-DR+% may not be a sensitive activation marker in children.

CD62L+CD45RA+ T-cells are naïve T-cells with high affinity for entry into secondary lymphoid tissues [9, 21–23]. The CD8+CD62L+CD45RA+% is dramatically reduced early in the course of HIV infection, resulting in undermined ability of the immune system to respond to novel antigens [9]. In the present study, the mean CD8+CD62L+CD45RA+% (naïve) increased over time in response to cART in the overall cohort, but was persistently higher in the CMV-naïve than in CMV+ groups, and increased at a slower rate in CMV+ compared to CMV-naïve group. This finding is consistent with previous reports of increased CD8+CD62L+CD45RA+% (naïve) following cART initiation in PHIV+ children [2] and suggests that the recovery of the naïve CD8+ subset may be derailed by CMV co-infection. Future studies are warranted to evaluate the clinical implications of this CMV-associated effect in PHIV+ children on suppressive cART, including overall morbidity and susceptibility to novel pathogens.

The CD95 molecule mediates activation-induced T-cell apoptosis [8]. The CD28 molecule, expressed on peripheral T cells and thymocytes, mediates an essential costimulatory signal following engagement of the T-cell receptor [24]. Increased proportions of CD28- effector memory T-cells are observed during normal aging and chronic immune stimulation [10]. The overall decrease in mean CD8+CD95+CD28-% (terminally differentiated) over the course of ACTG 366 trial tended to be less pronounced among CMV+ participants; an opposite trend was observed in the naïve CD8+CD95-CD28+%. Studies with adult populations [7] suggest that CMV infection is associated with accelerated immunosenescence. The present findings suggest that this may also be the case in PHIV+ infants, children and adolescents.

The observed impact of CMV infection on longitudinal trends was independent of treatment arm and inclusion of nelfinavir in the ART regimens, in spite of the in vitro activity of nelfinavir against CMV [16]. However, the differential effects of cART regimens on immune recovery were not the primary focus of this analysis and ACTG 366 randomization protocol was not based on inclusion of nelfinavir in cART regimens, so these results should not be considered definitive.

The loss of CMV-specific CMI commonly seen in patients with advanced HIV disease is deemed to contribute to CMV reactivation culminating in end-organ disease. We hypothesized that CMV-specific CMI, through its protective effect against CMV reactivation, may be associated with decreased CD8+ T-cell differentiation in our CMV and HIV co-infected subjects. However, the results of our sub-analysis imply that CMV-specific CMI may have significant adverse impact on the recovery of naïve CD8+ T-cell subsets. This finding may indicate that the CMI response to CMV is accompanied by CD8+ T-cell activation and consequent depletion of CD8+ naïve T cells. Future studies with adequate sample sizes should follow up on this preliminary finding, in order to better understand its mechanistic underpinnings, including the potential role of active CMV viremia vs. aviremic CMV positivity and the effect of CMV antivirals.

The present findings may have therapeutic implications. Similar to the valganciclovir trial in adults [5], adjunctive pharmacological suppression of CMV DNA may help optimize immune recovery in co-infected PHIV+ children as well. Yet, our findings suggest that, unlike HIV+ adults, in whom valganciclovir-induced CMV suppression was marked by decrease in CD8+CD38+HLA-DR+% (activated), in PHIV+ children the changes in CD8+CD62L+CD45RA+% (naïve), CD8+CD95-CD28+% (naïve) and CD8+CD95+CD28-% (terminally differentiated) may be more sensitive biomarkers of treatment response. Furthermore, the fact that CMV infection status, rather than CMV viremia status, was the factor that influenced normalization of selected CD8+ T-cell subsets in the present study suggests that treatments that simply decrease CMV viremia may have limited effect. This may be especially important to prevent the ongoing effect of CMV and HIV together on immunologic aging and long-term consequences such as diseases of aging and opportunistic infections.

This study has limitations. First, the cART regimens provided in ACTG 366 were not as potent as the ones available today and all participants had severe disease at study entry. Nevertheless, the findings do contribute in our understanding of the impact of two viral infections on immune changes and immunologic aging; and this is relevant to the very many PHIV+ children who are now young adults and may be impacted by their early CMV infection and potentially ongoing reactivation leading to premature senescence and diseases of aging. Furthermore, the findings are relevant to low-resource settings where most of the new perinatal HIV infections occur today, ART formularies are often limited, and infant CMV infection is highly prevalent. [25, 26] It would, thus, be important to replicate this analysis in a population of PHIV+ infants who have never had severe disease. Second, due to the exploratory nature of this analysis, there were no adjustments made for multiple testing, and thus we reported p-values less than 5% as significant. This allows maximum sensitivity in identifying potential effects of CMV co-infection on immune reconstitution. The reported p-values can provide readers an idea of strength of evidence for associations. Future adequately powered studies focused on the identified important markers are needed to confirm these results. Finally, the duration of follow-up was 40 weeks. There could be later discernable effects of CMV beyond 40 weeks following more sustained ART HIV VL suppression and decreases in HIV-related immune activation.

We conclude that longitudinal trends in percentages of CD8+CD62L+CD45RA+ (naïve), CD8+CD95-CD28+ (naïve), and CD8+CD95+CD28- (terminally differentiated) T-cells, following switch in cART, were significantly adversely affected by CMV co-infection in this cohort of PHIV+ children. Certain aspects of immune recovery in cART-treated PHIV+ children, specifically restoration of naïve CD8+ T-cell compartment and decrease of terminally differentiated CD8+ T-cell compartment might be derailed by CMV co-infection even after successful suppression of HIV viremia. These findings may have implications for adjunctive treatment strategies targeting CMV co-infection in PHIV+ children, especially those that are now adults or reaching young adulthood and may have accelerated immunologic aging and associated diseases.

## Supporting Information

S1 Table Selected CD4+ and CD8+ T-cell Percentages by CMV Status. (DOCX)
