# Supplementary material for: Effect of Cytomegalovirus Co-Infection on Normalization of Selected T-Cell Subsets in Children with Perinatally Acquired HIV Infection Treated with Combination Antiretroviral Therapy
Source: PLoS One. 2015 Mar 20;10(3):e0120474. doi: 10.1371/journal.pone.0120474 (PMC4368806; doi:10.1371/journal.pone.0120474)
Supplement: S1 Table — (DOCX) [file pone.0120474.s001.docx]

**Supporting Information Table S1**

| **Characteristic** | **Week** | **CMV+ viremic** | | **CMV+ aviremic** | | **CMV+ Subtotal** | | **CMV-naïve** | | **Total** | | **P-value^1^** |
| --- | --- | --- | --- | --- | --- | --- | --- | --- | --- | --- | --- | --- |
|  |  | **N** | **Mean (s.d.)** | **N** | **Mean (s.d.)** | **N** | **Mean (s.d.)** | **N** | **Mean (s.d.)** | **N** | **Mean (s.d.)** |  |
| CD4+CD38+HLA-DR+% | 0 | 15 | 18.0 (13.1) | 51 | 18.5 (20.8) | 66 | 18.4 (19.3) | 39 | 17.0 (17.1) | 105 | 17.9 (18.4) | 0.93 |
|  | 12 | 11 | 16.2 (14.3) | 42 | 8.9 (7.5) | 53 | 10.4 (9.6) | 25 | 13.0 (16.5) | 78 | 11.2 (12.2) | 0.14 |
|  | 20 | 10 | 14.6 (11.8) | 40 | 11.8 (13.3) | 50 | 12.3 (13.0) | 26 | 10.5 (15.2) | 76 | 11.7 (13.7) | 0.73 |
|  | 40 | 7 | 18.1 (23.0) | 36 | 8.7 (6.2) | 43 | 10.3 (11.0) | 20 | 6.2 (4.7) | 63 | 9.0 (9.6) | 0.02 |
| CD4+CD62L+CD45RA+% | 0 | 15 | 53.1 (29.6) | 52 | 52.7 (21.5) | 67 | 52.8 (23.3) | 39 | 52.6 (22.6) | 106 | 52.7 (22.9) | 1.00 |
|  | 12 | 12 | 50.2 (22.8) | 42 | 52.0 (20.0) | 54 | 51.6 (20.5) | 25 | 58.4 (24.8) | 79 | 53.7 (22.0) | 0.43 |
|  | 20 | 10 | 52.9 (21.6) | 40 | 54.0 (19.3) | 50 | 53.8 (19.6) | 26 | 53.3 (22.4) | 76 | 53.6 (20.4) | 0.99 |
|  | 40 | 7 | 60.3 (21.0) | 36 | 53.5 (17.1) | 43 | 54.6 (17.7) | 20 | 67.7 (12.9) | 63 | 58.7 (17.3) | 0.01 |
| CD4+CD95-CD28+% | 0 | 15 | 44.1 (26.1) | 52 | 45.8 (22.0) | 67 | 45.4 (22.8) | 40 | 48.5 (24.0) | 107 | 46.6 (23.2) | 0.78 |
|  | 12 | 11 | 47.9 (24.7) | 42 | 52.5 (15.3) | 53 | 51.6 (17.5) | 26 | 54.3 (22.9) | 79 | 52.5 (19.3) | 0.66 |
|  | 20 | 10 | 42.2 (27.5) | 40 | 50.4 (19.4) | 50 | 48.7 (21.2) | 27 | 55.6 (21.4) | 77 | 51.2 (21.4) | 0.23 |
|  | 40 | 7 | 48.7 (28.2) | 36 | 51.6 (16.4) | 43 | 51.1 (18.4) | 21 | 62.5 (17.8) | 64 | 54.8 (18.9) | 0.07 |
| CD4+CD95+CD28-% | 0 | 15 | 8.6 (14.0) | 52 | 4.8 (10.3) | 67 | 5.6 (11.2) | 40 | 6.6 (14.1) | 107 | 6.0 (12.3) | 0.53 |
|  | 12 | 11 | 3.5 (4.7) | 42 | 2.1 (2.5) | 53 | 2.4 (3.1) | 26 | 3.3 (7.5) | 79 | 2.7 (5.0) | 0.52 |
|  | 20 | 10 | 4.3 (5.3) | 40 | 2.6 (3.8) | 50 | 3.0 (4.1) | 27 | 3.4 (11.0) | 77 | 3.1 (7.3) | 0.79 |
|  | 40 | 7 | 8.4 (15.8) | 36 | 2.6 (2.7) | 43 | 3.5 (6.8) | 21 | 1.5 (2.3) | 64 | 2.9 (5.8) | 0.02 |
| CD4+CD62L-CD45RA+% | 0 | 15 | 2.5 (3.4) | 52 | 1.8 (2.1) | 67 | 2.0 (2.4) | 39 | 3.1 (4.7) | 106 | 2.4 (3.5) | 0.20 |
|  | 12 | 12 | 2.0 (2.9) | 42 | 1.0 (1.0) | 54 | 1.3 (1.6) | 25 | 3.2 (7.3) | 79 | 1.9 (4.4) | 0.15 |
|  | 20 | 10 | 2.2 (3.1) | 40 | 2.2 (2.7) | 50 | 2.2 (2.8) | 26 | 2.9 (7.5) | 76 | 2.4 (4.9) | 0.83 |
|  | 40 | 7 | 3.3 (5.0) | 36 | 1.4 (1.7) | 43 | 1.7 (2.5) | 20 | 1.8 (3.1) | 63 | 1.7 (2.7) | 0.24 |
| CD8+CD38+HLA-DR+% | 0 | 15 | 41.4 (20.9) | 51 | 41.2 (21.0) | 66 | 41.2 (20.8) | 40 | 34.0 (17.4) | 106 | 38.5 (19.8) | 0.19 |
|  | 12 | 12 | 32.6 (19.3) | 42 | 26.4 (19.3) | 54 | 27.8 (19.3) | 26 | 20.7 (18.7) | 80 | 25.5 (19.3) | 0.19 |
|  | 20 | 10 | 36.6 (18.2) | 39 | 27.0 (20.4) | 49 | 29.0 (20.2) | 27 | 19.4 (15.1) | 76 | 25.6 (19.0) | 0.04 |
|  | 40 | 7 | 32.1 (18.9) | 36 | 30.6 (20.4) | 43 | 30.8 (19.9) | 21 | 17.6 (12.1) | 64 | 26.5 (18.7) | 0.03 |
| CD8+CD62L+CD45RA+% | 0 | 15 | 28.9 (13.6) | 52 | 30.6 (15.7) | 67 | 30.2 (15.2) | 38 | 37.1 (17.8) | 105 | 32.7 (16.5) | 0.11 |
|  | 12 | 12 | 36.0 (14.1) | 42 | 35.6 (19.9) | 54 | 35.7 (18.7) | 24 | 54.7 (20.8) | 78 | 41.5 (21.1) | <.001 |
|  | 20 | 10 | 36.6 (24.5) | 40 | 37.2 (21.2) | 50 | 37.1 (21.6) | 25 | 51.6 (18.9) | 75 | 41.9 (21.7) | 0.02 |
|  | 40 | 7 | 41.4 (20.8) | 36 | 39.3 (21.4) | 43 | 39.6 (21.1) | 19 | 61.5 (17.9) | 62 | 46.3 (22.5) | 0.001 |
| CD8+CD95-CD28+% | 0 | 15 | 11.9 (11.6) | 52 | 14.3 (13.7) | 67 | 13.8 (13.2) | 40 | 19.6 (13.0) | 107 | 16.0 (13.4) | 0.08 |
|  | 12 | 11 | 22.1 (18.6) | 42 | 18.5 (16.8) | 53 | 19.3 (17.1) | 25 | 28.9 (19.4) | 78 | 22.4 (18.3) | 0.08 |
|  | 20 | 10 | 20.4 (19.8) | 40 | 19.9 (18.1) | 50 | 20.0 (18.3) | 27 | 33.3 (17.9) | 77 | 24.7 (19.1) | 0.01 |
|  | 40 | 7 | 21.1 (13.5) | 36 | 21.7 (18.3) | 43 | 21.6 (17.4) | 21 | 40.2 (19.3) | 64 | 27.7 (20.0) | 0.001 |
| CD8+CD95+CD28-% | 0 | 15 | 49.9 (20.9) | 52 | 48.7 (19.2) | 67 | 49.0 (19.4) | 40 | 39.3 (17.6) | 107 | 45.4 (19.2) | 0.04 |
|  | 12 | 11 | 45.4 (20.3) | 42 | 38.0 (20.4) | 53 | 39.5 (20.4) | 25 | 25.2 (14.8) | 78 | 34.9 (19.9) | 0.005 |
|  | 20 | 10 | 50.7 (23.2) | 40 | 42.4 (23.6) | 50 | 44.0 (23.5) | 27 | 22.1 (14.9) | 77 | 36.3 (23.3) | <.001 |
|  | 40 | 7 | 42.7 (22.3) | 36 | 43.3 (21.8) | 43 | 43.2 (21.6) | 21 | 20.1 (11.3) | 64 | 35.6 (21.7) | <.001 |
| CD8+CD62L-CD45RA+% | 0 | 15 | 24.7 (13.8) | 52 | 25.3 (16.9) | 67 | 25.2 (16.1) | 38 | 17.2 (12.2) | 105 | 22.3 (15.3) | 0.04 |
|  | 12 | 12 | 24.6 (12.0) | 42 | 21.6 (14.0) | 54 | 22.2 (13.5) | 24 | 14.3 (8.4) | 78 | 19.8 (12.7) | 0.03 |
|  | 20 | 10 | 18.2 (9.9) | 40 | 22.3 (15.5) | 50 | 21.5 (14.5) | 25 | 12.4 (5.2) | 75 | 18.4 (12.9) | 0.01 |
|  | 40 | 7 | 25.6 (6.1) | 36 | 19.9 (14.0) | 43 | 20.8 (13.1) | 19 | 11.8 (7.9) | 62 | 18.1 (12.4) | 0.01 |

^1^Comparison of CMV+ viremic, CMV+ aviremic, and CMV-naïve groups using Analysis of Variance tests
